# Supplementary material for: Fsr quorum sensing system restricts biofilm growth and activates inflammation in enterococcal infective endocarditis
Source: bioRxiv. 2025 Feb 7:2025.02.07.636843. Preprint. [Version 1] doi: 10.1101/2025.02.07.636843 (PMC11839028; doi:10.1101/2025.02.07.636843)
Supplement: Supplement 1 [file media-1.pdf]

SUPPLEMENTARY INFORMATION

Fsr quorum sensing system restricts biofilm growth and activates inflammation in enterococcal infective endocarditis

Haris ANTYPAS[1,*], Verena SCHMIDTCHEN[2], Willy Isao STAIGER[2], Yanhong LI[3,4], Rachel Jing Wen TAN [1], Kenneth Kok Fei NG[1], Cheryl Jia Yi NEO[1], Shalome Meera RADHESH[1], Frederick Reinhart TANOTO[1], Ronni Anderson Gonçalves DA SILVA[1,5], Cristina Colomer WINTER[6], Caroline MANZANO[6], Jun Jie WONG[1], Kevin PETHE[1,7,8], Barbara HASSE[2], Silvio Daniel BRUGGER[2], Siu Ling WONG[7,9], Daria VAN TYNE[3], Annelies S. ZINKERNAGEL[2], Kimberly A. KLINE[1,5,6,10,*]

[1]Singapore Centre for Environmental Life Sciences Engineering, School of Biological Sciences, Nanyang Technological University, Singapore

[2]Division of Infectious Diseases, University Hospital Zurich, CH-8091 Zurich, Switzerland

[3] Department of Medicine, University of Pittsburgh, PA, USA

[4] School of Medicine, Tsinghua University, Beijing, China

[5]Singapore-MIT Alliance for Research and Technology Centre, Singapore

[6]Department of Microbiology and Molecular Medicine, University of Geneva, Switzerland

[7]Lee Kong Chian School of Medicine, Nanyang Technological University, Singapore

[8]National Center for Infectious Diseases (NCID), 308442, Singapore

[9] Tan Tock Seng Hospital, Singapore

[10]Lead Contact

[*]Correspondence: Haris.Antypas@ntu.edu.sg, Kimberly.Kline@unige.ch

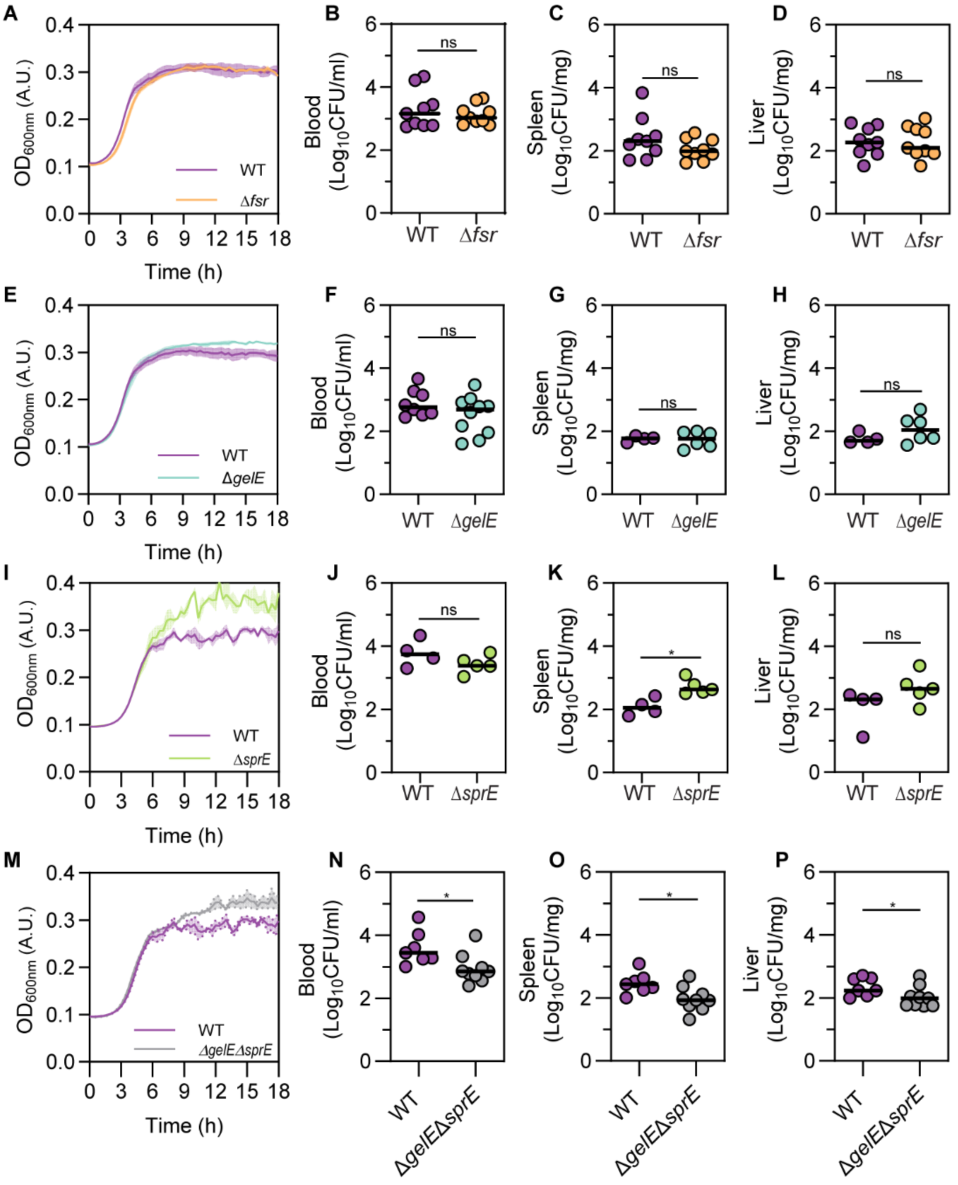

Fig. S1. Growth and systemic spread of *E. faecalis* in the absence of Fsr QS system, *gelE*, and *sprE* in IE at 72 hpi. A, E, I, M. Growth curves in BHIS at 37°C under aerobic conditions without shaking. Error = SEM represented as shaded region. N = 3 independent experiments for (A) and (E), N = 1 for (I) and (M). **B-D, F-H, J-L, N-P**. Median of blood, liver, and spleen CFU at 72 hpi. n = 9 animals per group from N = 2 is shown for (B-D), n = 8 - 10 animals per group from N = 2 for (F), n = 4 – 6 from N = 1 for (G-H), n = 4 – 5 from N = 1 for (J-L), n = 7 – 9 from N = 2 for (N-P). Statistical significance in median difference was assessed by applying a Mann-Whitney test; * = $p < 0.05$, ns = not significant.

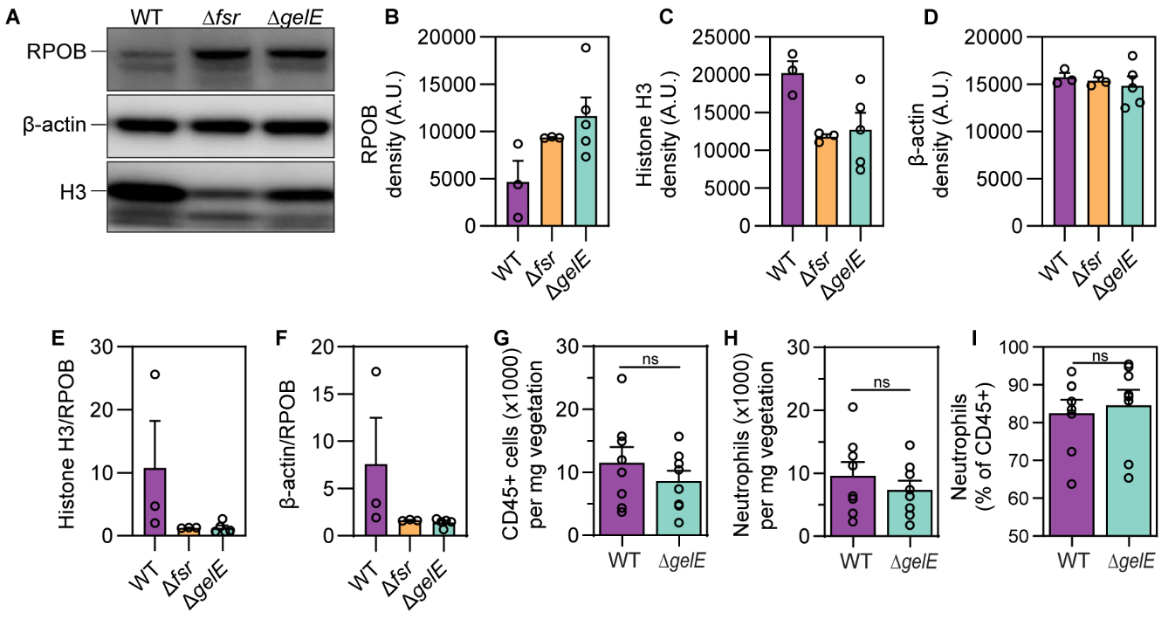

Fig. S2. **Increased bacterial load in Δ*fsr*- and Δ*gelE*-infected vegetations does not promote a further increase in host cell infiltration**. **A-D.** *E. faecalis* RPOB, and rat histone H3 and β-actin detection with western blotting in lysates from WT, Δ*fsr*-, and Δ*gelE*-infected vegetations harvested at 72 hpi (A), and protein band density quantification with ImageJ (B-D). RPOB levels (B) corresponding to bacterial abundance are higher in Δ*fsr*- and Δ*gelE*-infected vegetations compared to WT. Histone H3 levels (C) corresponding to nucleated host cell abundance and β-actin levels (D) corresponding to both nucleated host cells and anucleate platelets are similar across all vegetations. Representative bands are shown in A. Samples were harvested from n = 3-5 animals per group from N = 1 independent experiment. Error = SEM. **E-F.** Histone H3 and β-actin levels normalized by RPOB levels, reflecting the relative abundance of host cells to bacteria. Analysis is based on results from (A-D). **G-I.** Leukocyte (CD45+) (G) and neutrophil (H) absolute quantification, and neutrophil relative quantification (I) at 72 hpi vegetations using flow cytometry. Neutrophils (% of CD45+ cells) were determined based on the number of CD45+ RP-1+ events of the total CD45+ events. Mean with SEM is shown from n = 8 animals per group from N = 2 independent experiments. Statistical significance was assessed with a t-test; ns = not significant

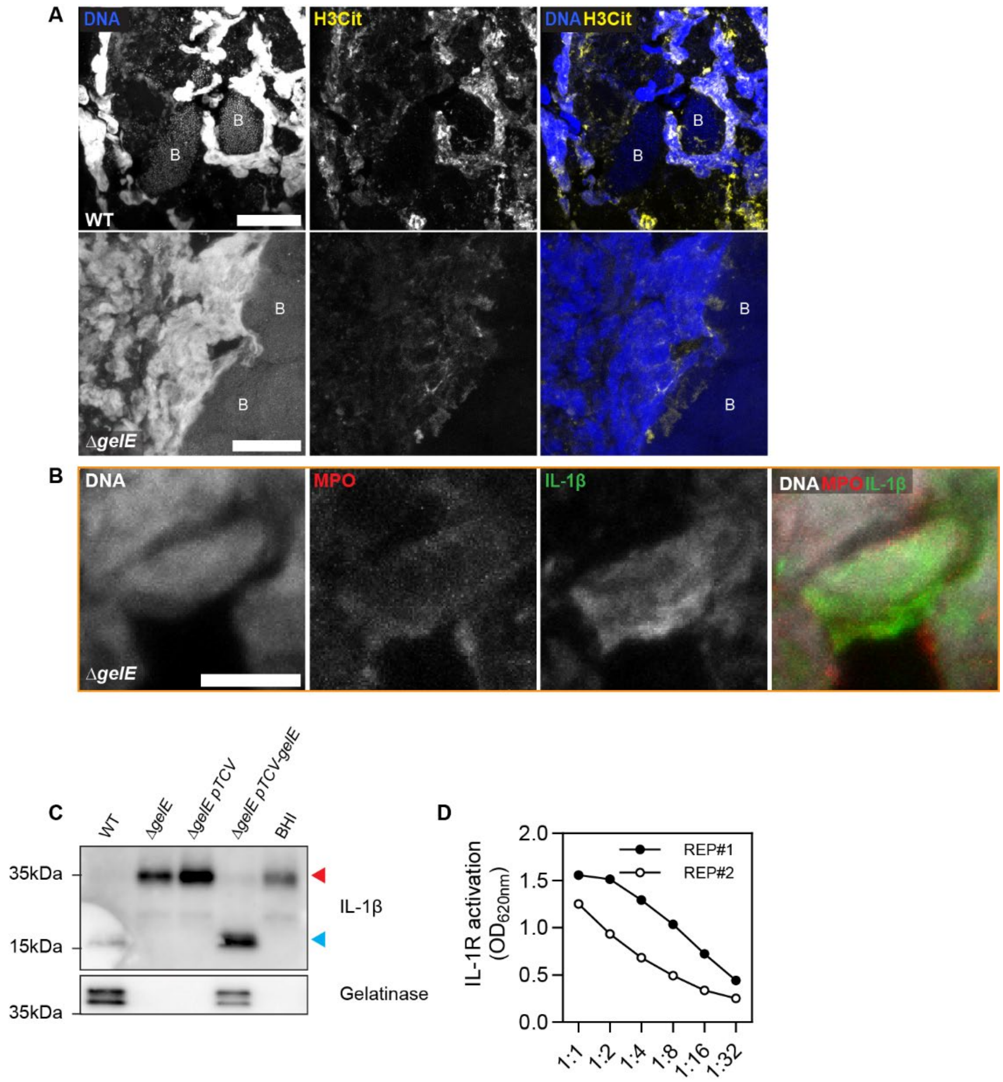

Fig. S3. *E. faecalis* gelatinase cleaves and activates IL-1β. A. Neutrophils undergoing NETosis at the interface with biofilm (B), evidenced by their decondensed nuclei colocalizing with citrullinated histone H3 (H3Cit) in WT- and Δ*gelE*-infected vegetations at 72 hpi. Representative Z-projection captured with LSCM from n =3 animals per group from N = 1 is shown. DNA is stained with DAPI. Scale = 20 µm. **B.** Inset (orange) from Fig. 5A (Δ*gelE* panel) showing IL-1β colocalizing with a decondensed nucleus of an incoming neutrophil. Colocalization with myeloperoxidase (MPO) indicates that the nucleus belongs to a neutrophil. Scale = 5 µm. **C.** Complementation of Δ*gelE* with pTCV-Ptet-*gelE* (Δ*gelE* pTCV-*gelE*) restored secretion of gelatinase in the supernatant and cleaving of pro-IL-1β (blue arrowhead) to a 17 kDa fragment (red arrowhead). **D.** Activation of HEK-Blue IL-1R reporter cells in the presence of 2-fold serial dilutions of supernatants harvested from OG1RF WT incubated with pro-IL-1β for 18 h. N = 2.

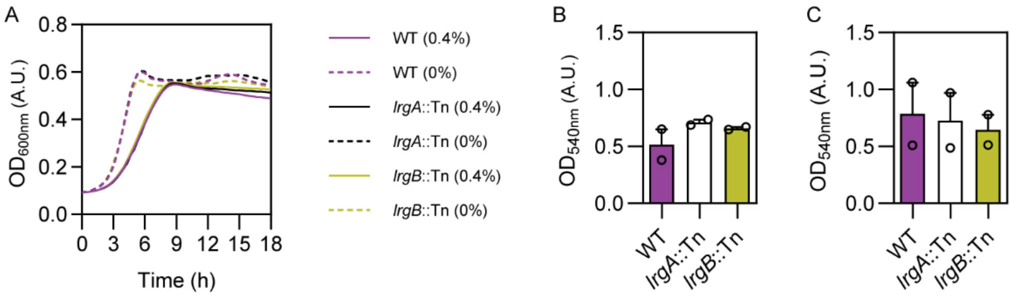

Fig. S4. *lrgAB* is not involved in bacterial sensitivity to Triton X-100 and biofilm formation. A. Growth curves of *E. faecalis* strains in BHI with 0.4 or 0 % Triton X-100 at 37°C under aerobic conditions without shaking. Mean of 3 technical replicates from N = 1 is shown. **B-C.** Biofilm formation at 24 h on tissue culture-treated (B) and uncoated polystyrene (C) 96-well plates assessed with the crystal violet assay. Mean of N = 2 is shown.

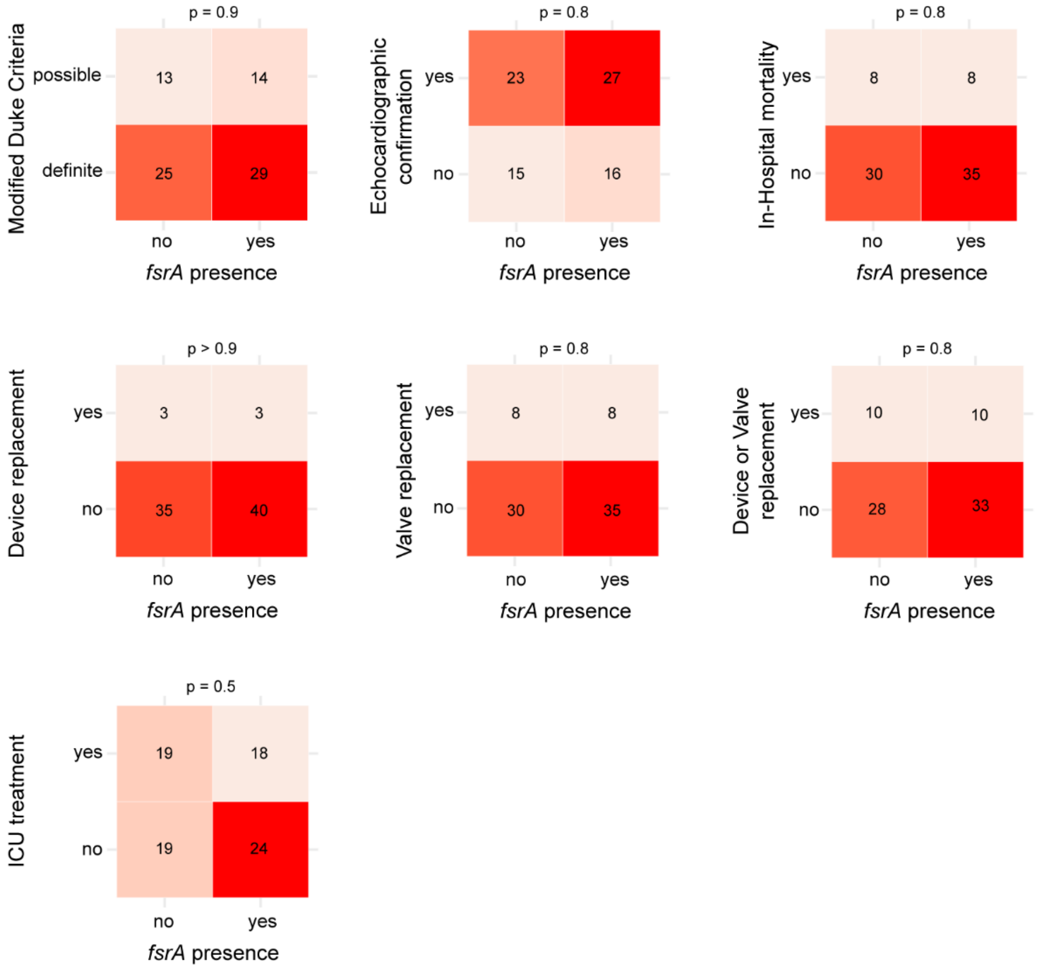

Fig. S5. Relationship between *fsr* presence and clinical parameters of IE. Contingency tables of the association of *fsrA* presence with different clinical parameters are shown. Statistical significance was assessed by Fisher's exact test.

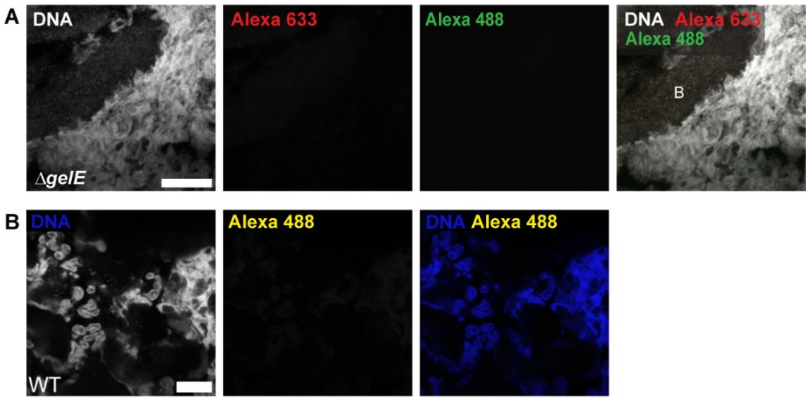

Fig. S6. Secondary antibody only controls. A. Secondary antibody only control performed on a tissue section consecutive to the section shown in Fig. 5A (Δ*gelE* panel). Z-projection of images captured with LSCM is shown, stained for DNA, goat anti-rabbit IgG Alexa 488, and goat anti-mouse IgG1 Alexa 633. B = biofilm, scale = 20 µm. **B.** Z-projections of images captured from WT-infected vegetations at 72 hpi captured with LSCM, stained for DNA and goat anti-rabbit IgG Alexa 488. Representative image of neutrophils undergoing NETosis from N=3 is shown. Scale = 20 µm.

Table S1. MIC of ampicillin for *E. faecalis* OG1RF WT, *lrgA*::Tn, and *lrgB*::Tn strains

	MIC (μg/ml)		
	**Rep#1**	**Rep#2**	**Rep#3**
**WT**	0.5	1	2
***lrgA*::Tn**	0.5	1	2
***lrgB*::Tn**	0.5	1	2

Table S2. MIC of gentamicin for *E. faecalis* OG1RF WT and Δ*fsr* strains

	MIC (μg/ml)		
	**Rep#1**	**Rep#2**	**Rep#3**
**WT**	32	16	32
**Δ*fsr***	32	16	32

Table S3. ***E. faecalis*** **strains and plasmids used in** ***in vitro*** **and** ***in vivo*** **assays**

Strain	Description	Reference
OG1RF	*Enterococcus faecalis, Rif^R, Fus^R*	[1]
Δ*fsr*	OG1RF *fsrABDC* deletion	This work
Δ*gelE*	OG1RF *gelE* deletion	[2]
Δ*sprE*	OG1RF *sprE* deletion	This work
Δ*gelE*Δ*sprE*	OG1RF Δ*gelE*Δ*sprE* deletion	This work
Δ*gelE*::*gelE*^{E352A}	OG1RF Δ*gelE*::*gelE*^{E352A} (proteolytically inactive gelatinase)	This work
Δ*gelE* pTCV-P_{tet}	*gelE* deletion mutant with empty expression vector	This work
Δ*gelE* pTCV-P_{tet}::*gelE*	*gelE* deletion mutant with constitutively expressed *gelE*	This work
sprE::Tn	OG1RF, Tn insertion at site 1586796, Cm^R	[3]
lrgA::Tn	OG1RF, Tn insertion at site 2597437, Cm^R	[3]
lrgB::Tn	OG1RF, Tn insertion at site 2597099, Cm^R	[3]
pGCP213	Gram-positive, temperature-sensitive shuttle vector for allelic replacement	[4]
pTCV-P_{tet}	Low-copy shuttle vector with constitutive P_{tet} promoter for expression; Kan^R, Erm^R	[5,6]
pTCV-P_{tet}::*gelE*	Complementation plasmid for *gelE* expression under constitutive P_{tet} promoter; Kan^R, Erm^R	This work

Table S4. Oligonucleotides used in this study

Oligo	Sequence (5'-3')	Description
fsrA_F	AGCAACCTCAAATCCTGCCT	qPCR
fsrA_R	TACAAGTGGCACACCAGGAC	qPCR
Primer 2	ATGAGTGAACAAATGGCTATTTA[1]	FW primer, *fsrA* detection in clinical isolates
Primer 3	CTAAGTAAGAAATAGTGCCTTGA[1]	RV primer, *fsrA* detection in clinical isolates
fsrB_F	AGACCTTGGATGACGAGACCG	qPCR
fsrB_R	GGTATGCGCCACAAGGAACA	qPCR
fsrC_F	TGCACTGTTTTCAATCGCGT	qPCR
fsrC_R	ACCGCAAAGCAAGCAAAACT	qPCR
gelE_F	ACAAGATGGGCATCCCTCGA	qPCR
gelE_R	TCAAGCGCCATCACTAGCGA	qPCR
sprE _F	ATTGCGGTAGTGACTGTCGG	qPCR
sprE_R	CGACCATTGCGTGTGGTTTT	qPCR
entV_F	AGCTGCACAAAAGAAAGCCTG	qPCR
entV_R	TAGCCCACATTGAACTGCCC	qPCR
RS04585_F	ACTTGGATAGATTCAGGAGAACTTG	qPCR
RS04585_R	TGGGTATTGCATAGAACATGGC	qPCR
recA_F	GCGGCTGTTCCACCATTTCG	qPCR
recA_R	GTTGCATTGGGCGTAGGTGG	qPCR
dnaB_F	CGTGGTGAAGGAGAAGACGGT	qPCR
dnaB_R	TCCTCGGCGAAAGCGAAGAA	qPCR
fsrABDC_del_1	ATTGCTTACTCGAGTGGCGTGAC[2]	*fsrABDC* upstream-F for Δ*fsrABDC* mutant construction
fsrABDC_del_2	TTCGTTAACAACTTTTTTGTTCACTCATCCCTTTCTC	*fsrABDC* upstream-R for Δ*fsrABDC* mutant construction

fsrABDC_del_3	GGATGAGTGAACAAAAAAGTTGTTAACGAATGAATTTG	*fsrABDC* downstream-F for Δ*fsrABDC* mutant construction
fsrABDC_del_4	CCGTTTGCTTTTCAAGCTTAAGATGCTT[2]	*fsrABDC* downstream-R for Δ*fsrABDC* mutant construction
sprE-del-1	GTTGACCGAAAAACAGGAATTCGAAATTTA[2]	*sprE* upstream-F for Δ*sprE* mutant construction
sprE-del-2	CACAGCGGATAAACGGATCATGCCACTCCTTATCC	*sprE* upstream-R for Δ*sprE* mutant construction
sprE-del-3	TAAGGAGTGGCATGATCCGTTTATCCGCTGTGCCAGC	*sprE* downstream-F for Δ*sprE* mutant construction
sprE_del_4 & gelE_del_4	AAAGAAAGAAAGCTTGTCACAGATAAAACG[2]	*sprE* downstream-R for Δ*sprE* mutant construction
gelE_del_1	GTGTCCAAGCCGAATTCGATTTTAG[2]	*gelE* upstream-F for Δ*gelE* Δ*sprE* mutant construction
gelE_del_2	GCACAGCGGATAAACGTTCCAACAAAGATGCCTGT	*gelE* upstream-R for Δ*gelE* Δ*sprE* mutant construction
gelE_del_3	TACAGGCATCTTTGTTGGAACGTTTATCCGCTGTGCCAGC	*gelE* downstream-*F for* Δ*gelE* Δ*sprE* mutant construction
gelE_ins_1	gatgcatgctcgagcGAATTGAAAATGTTCGCTATCTC[3]	*gelE* upstream-F for *gelE* chromosomal insertion
gelE_ins_2	GAATAAACTTGTTCTTC**C**GCGGC[4]	*gelE* upstream-R with silent mutation for *gelE* chromosomal insertion
gelE_ins_3	GTAGCCGC**G**GAAGAACAAG[4]	*gelE* downstream-F with silent mutation for *gelE* chromosomal insertion
gelE_ins_4	taccgagctcggatcGACGATCGTTTTGTTTGC[3]	*gelE* downstream-R for *gelE* chromosomal insertion
pGCP213_F	GCTCGAGCATGCATCTAGAGG	pGCP213 inverse PCR-F
pGCP213_R	GATCCGAGCTCGGTACCAAG	pGCP213 inverse PCR-R
gelE_E352A_SDM_F	CAGGTGCCTTGAATG**C**ATCTTATTCTG[4]	*gelE*(E352A) site directed mutagenesis-F
gelE_E352A_SDM_R	CAGAATAAGAT**G**CATTCAAGGCACC[4]	*gelE*(E352A) site-directed mutagenesis SDM-R
gelE_pTCV-Ptet_F	ATGCCTATGGGATCCGAGGATAAAGCAATACTTTTGTTGG	*gelE* insert-F for plasmid complementation
gelE_pTCV-Ptet_R	CTTGCATGCCTGCAGGAATTTTTTTCATTCATTGACCAG	*gelE* insert-R for plasmid complementation

[1]Nakayama J, Kariyama R, Kumon H, 2002

[2]Underlined nucleotides indicate restriction site

[3]15bp overhang of homologous region for InFusion (Gibson assembly) in lowercase

[4]Point mutations for SDM annotated in bold

References

1. Dunny, G.M., Brown, B.L., and Clewell, D.B. (1978). Induced cell aggregation and mating in Streptococcus faecalis: evidence for a bacterial sex pheromone. Proc Natl Acad Sci U S A *75*, 3479–3483. https://doi.org/10.1073/PNAS.75.7.3479.

2. Thomas, V.C., Hiromasa, Y., Harms, N., Thurlow, L., Tomich, J., and Hancock, L.E. (2009). A fratricidal mechanism is responsible for eDNA release and contributes to biofilm development of Enterococcus faecalis. Mol Microbiol *72*, 1022. https://doi.org/10.1111/J.1365-2958.2009.06703.X.

3. Kristich, C.J., Nguyen, V.T., Le, T., Barnes, A.M.T., Grindle, S., and Dunny, G.M. (2008). Development and Use of an Efficient System for Random mariner Transposon Mutagenesis To Identify Novel Genetic Determinants of Biofilm Formation in the Core Enterococcus faecalis Genome. Appl Environ Microbiol *74*, 3377. https://doi.org/10.1128/AEM.02665-07.

4. Nielsen, H. V., Guiton, P.S., Kline, K.A., Port, G.C., Pinkner, J.S., Neiers, F., Normark, S., Henriques-Normark, B., Caparon, M.G., and Hultgren, S.J. (2012). The metal ion-dependent adhesion site motif of the Enterococcus faecalis EbpA pilin mediates pilus function in catheter-associated urinary tract infection. mBio *3*. https://doi.org/10.1128/MBIO.00177-12/SUPPL_FILE/MBO004121300ST4.PDF.

5. Tan, C.A.Z., Chong, K.K.L., Yeong, D.Y.X., Ng, C.H.M., Ismail, M.H., Yap, Z.H., Khetrapal, V., Tay, V.S.Y., Drautz-Moses, D.I., Ali, Y., et al. (2024). Purine and carbohydrate availability drive Enterococcus faecalis fitness during wound and urinary tract infections. mBio *15*. https://doi.org/10.1128/mbio.02384-23.

6. Danne, C., Guérillot, R., Glaser, P., Trieu-Cuot, P., and Dramsi, S. (2013). Construction of isogenic mutants in Streptococcus gallolyticus based on the development of new mobilizable vectors. Res Microbiol *164*, 973–978. https://doi.org/10.1016/J.RESMIC.2013.09.002.
